# Supplementary material for: cGAS-STING dependent type I IFN reduces Leptospira interrogans renal colonization in mice
Source: PLoS Pathog. 2026 Jan 7;22(1):e1013250. doi: 10.1371/journal.ppat.1013250 (PMC12795460; doi:10.1371/journal.ppat.1013250)
Supplement: S1 Fig — (A) Dose dependent induction of IFNβ was quantified by ELISA in culture supernatants of WT BMDMs infected with L. interrogans (MOI 1, 10, 100) for 4, 6, and 8h. Data are pooled from two independent experiments and expressed as the mean ± SD. (B) Quantification of IFNβ levels by ELISA in culture supernatants of WT BMDM infected with live or heat-killed L. interrogans (MOI 100) at 6h post-infection. Data are pooled from three independent experiments. Statistical significance was calculated by unpaired Student’s t test. (C) Quantification of IFNβ levels by ELISA in culture supernatants of WT BMDM infected with L. interrogans Fiocruz L1-130 (MOI 100) or L. biflexa Patoc 1 (MOI 100) at 4, 6, and 8h post-infection. Statistical significance calculated by two-way ANOVA. ns = non-significant. (DOCX) [file ppat.1013250.s001.docx]

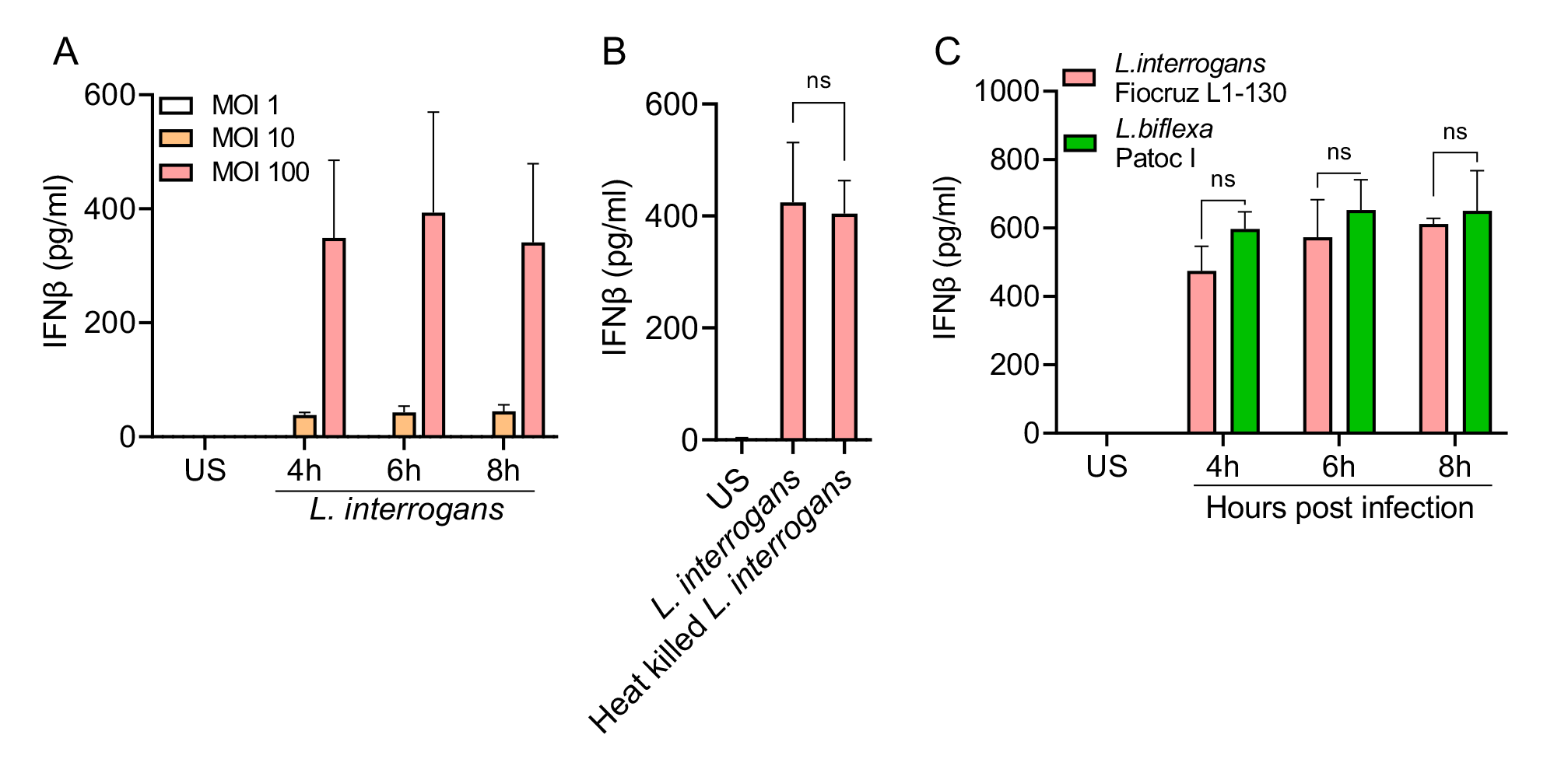


**S1 Fig. Analysis of IFNβ production from BMDM infected with *Leptospira.*** (A) Dose dependent induction of IFNβ was quantified by ELISA in culture supernatants of WT BMDMs infected with *L. interrogans* (MOI 1, 10, 100) for 4, 6, and 8h. Data are pooled from two independent experiments and expressed as the mean ± SD. (B) Quantification of IFNβ levels by ELISA in culture supernatants of WT BMDM infected with live or heat-killed *L. interrogans* (MOI 100) at 6h post-infection. Data are pooled from three independent experiments. Statistical significance was calculated by unpaired Student’s *t* test. (C) Quantification of IFNβ levels by ELISA in culture supernatants of WT BMDM infected with *L. interrogans* Fiocruz L1-130 (MOI 100) or *L. biflexa* Patoc 1 (MOI 100) at 4, 6, and 8h post-infection. Statistical significance calculated by two-way ANOVA. ns=non-significant.
