# Supplementary material for: cGAS-STING dependent type I IFN reduces Leptospira interrogans renal colonization in mice
Source: PLoS Pathog. 2026 Jan 7;22(1):e1013250. doi: 10.1371/journal.ppat.1013250 (PMC12795460; doi:10.1371/journal.ppat.1013250)
Supplement: S2 Fig — Quantification of immunofluorescence images of bacteria bound or total bacteria (bound and internalized) in WT, Cgas-/- and Sting1gt/gt BMDMs at 3h post infection with L. interrogans (MOI 100). Data are pooled from three frames per experiment from three independent experiments. Statistical significance was calculated by two-way ANOVA. ns = non-significant. (DOCX) [file ppat.1013250.s002.docx]

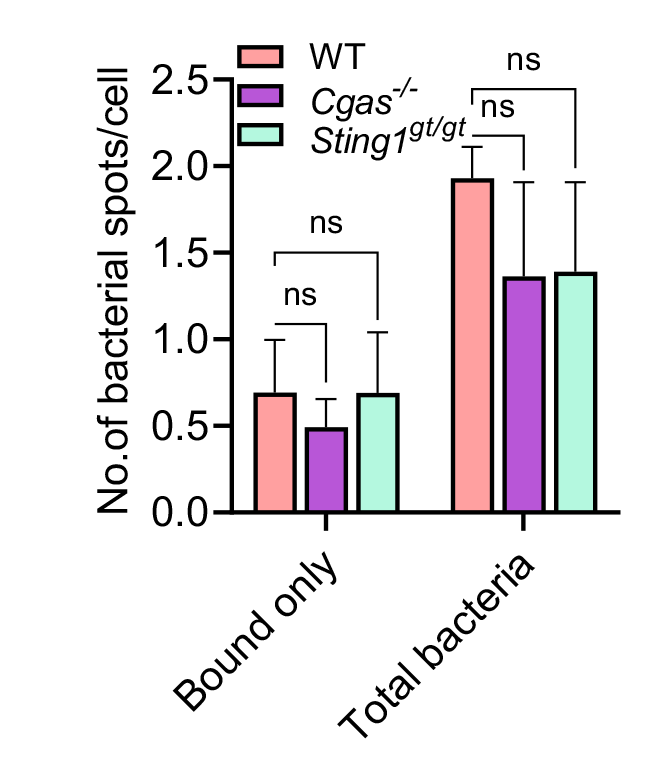


**S2 Fig. Analysis of *L. interrogans* binding and internalization by BMDM.** Quantification of immunofluorescence images of bacteria bound or total bacteria (bound and internalized) in WT, *Cgas^-/-^* and *Sting1^gt/gt^* BMDMs at 3h post infection with *L. interrogans* (MOI 100)*.* Data are pooled from three frames per experiment from three independent experiments. Statistical significance was calculated by two-way ANOVA. ns=non-significant.
