# Supplementary material for: cGAS-STING dependent type I IFN reduces Leptospira interrogans renal colonization in mice
Source: PLoS Pathog. 2026 Jan 7;22(1):e1013250. doi: 10.1371/journal.ppat.1013250 (PMC12795460; doi:10.1371/journal.ppat.1013250)
Supplement: S1 Table — (DOCX) [file ppat.1013250.s003.docx]

S1 Table: Primers used for qPCR

| **Mouse Primers** | **Sequence (5'-3')** |
| --- | --- |
| *Ifna* (F) | CCT GAG AGA GAA GAA ACA CAG CC |
| *Ifna* (R) | GGC TCT CCA GAC TTC TGC TCT G |
| *Ifnb* (F) | GCT CCT GGA GCA GCT GAA TG |
| *Ifnb* (R) | CGT CAT CTC CAT AGG GAT CTT GA |
| *Ifit 1* (F) | CAA GGC AGG TTT CTG AGG AG |
| *Ifit 1* (R) | GAC CTG GTC ACC ATC AGC AT |
| *Ifit 3* (F) | TTC CCA GCA GCA CAG AAA C |
| *Ifit 3* (R) | AAA TTC CAG GTG AAA TGG CA |
| *Ifi 44* (F) | CTG ATT ACA AAA GAA GAC ATG ACA GAC |
| *Ifi 44* (R) | AGG CAA AAC CAA AGA CTC CA |
| *Zbp 1* (F) | TCA AAG GGT GAA GTC ATG GA |
| *Zbp 1* (R) | GTG GAG TGG CTT CAG AGC TT |
| *Tnfa* (F) | GGT GCC TAT GTC TCA GCC TCT T |
| *Tnfa* (R) | GCC ATA GAA CTG ATG AGA GGG AG |
| *Ppia* (F) | GAG CCA CTC ACC TGA TGC TTA |
| *Ppia* (R) | GGC AAT GAA AAT GCT ACC ACC TT |
| *Actin b* (F) | CGA GGT ATC CTG ACC CTG AA |
| *Actin b* (R) | GGT GTG GTG CCA GAT CTT CT |
| **Human primers** | **Sequence (5'-3')** |
| *Ifnb* (F) | AAA CTC ATG AGC AGT CTG CA |
| *Ifnb* (R) | AGG AGA TCT TCA GTT TCG GAG G |
| *Actin b* (F) | CAC CAT TGG CAA TGA GCG GTT C |
| *Actin b* (R) | AGG TCT TTG CGG ATG TCC ACG T |
| **Bacteria primers** | **Sequence (5'-3')** |
| *lipL32* (F) | AAG CAT TAC CGC TTG TGG TG |
| *lipL32* (R) | GAA CTC CCA TTT CAG CGA TT |
