# Supplementary material for: cGAS-STING dependent type I IFN reduces Leptospira interrogans renal colonization in mice
Source: PLoS Pathog. 2026 Jan 7;22(1):e1013250. doi: 10.1371/journal.ppat.1013250 (PMC12795460; doi:10.1371/journal.ppat.1013250)
Supplement: Attachment — Submitted filename: Response to Reviewers .pdf [file ppat.1013250.s005.pdf]

Response to Reviewers (PPATHOGENS-D-25-01317)

Additional Editor Comments:
"…the authors need to clarify some of the experimental details. For instance, with regards to replicates, Figure 1 and 2 state that "Data pooled from three experiments" – how many technical replicates would this be per experiment?"

We have clarified these details in the methods section. Each biological replicate was done as a technical duplicate or triplicate. The three biological replicates were pooled and expressed as the mean ± SEM.

"Furthermore, some of the findings do not support the conclusions made by the authors and it would be important to moderate some of their assertions."

We have carefully revised the manuscript and have reworded the noted sections to better reflect the presented data.

Reviewers' Comments:

Part I - Summary

Reviewer #1: The manuscript by Gupta et al. describes in vivo and in vitro experiments to demonstrate that cGAS-STING-driven type I IFN is required for the host defense against L. interrogans. Despite the interesting results showing a potential role of this pathway on overall leptospirosis infection, the methods and results presented here are not sufficient to eliminate the bias and do not support the conclusions of the authors. This pathway might have a role on the innate immune response against leptospires, but the results presented here are not enough to show that they are essential or even important to control leptospiral infection.

We appreciate the thoughtful comments from the reviewer. We have added additional in vivo data assessing renal colonization at day 14 post-infection in mice intraperitoneally infected with *L. interrogans* serovar Copenhageni strain Fiocruz L1-130. Consistent with our previous findings we observe significantly higher bacterial burdens in the kidneys of *Ifnar1*$^{-/-}$ (Fig 3G) and *Sting*$^{gt/gt}$ (Fig 5E) mice. Taken together these data support a role for cGAS-STING in limiting renal colonization by *L. interrogans* in mice. As mice serve as a reservoir species for *L. interrogans*, infection in mice is asymptomatic and results in renal colonization and urinary shedding. Even though we did not observe increased mortality in *Ifnar1*$^{-/-}$ (Fig 3D) and *Sting*$^{gt/gt}$ (Fig 5B) mice infected with *L. interrogans*, the finding that there are increased renal bacterial burdens in *Ifnar1*$^{-/-}$ and *Sting*$^{gt/gt}$ mice is highly relevant to understanding the innate immune pathways that control renal colonization.

Reviewer #2: This manuscript presents a novel approach to targeting a major innate immune signaling pathway—cGAS-STING—which is central to sensing extracellular dsDNA and triggering downstream type I interferon responses. While the study is well-designed and employs a variety of approaches, the current experimental evidence partially supports the major claims. Additional experiments—particularly involving cGAS-deficient models, increasing biological replicates, and further mechanistic insights would further substantiate the conclusions.

We appreciate the concerns of the reviewer and have added additional information regarding scientific rigor and statistical analysis. We have also added additional new data to examining bacterial burdens in the spleen and kidney at day 14 post-infection with *L. interrogans* via the intraperitoneal route (Fig 3G and 5E). Unfortunately performing in vivo studies using *Cgas*$^{-/-}$ mice was not possible in the timeframe required for resubmission of this study as we do not have an active breeding colony of *Cgas*$^{-/-}$ mice in our vivarium. Given that STING is downstream of cGAS and based on our in vitro findings with macrophages from *Cgas*$^{-/-}$ and *Sting*$^{gt/gt}$ mice (Fig 4), we feel that using *Ifnar1*$^{-/-}$ and *Sting*$^{gt/gt}$ mice in vivo is sufficient to conclude that this pathway contributes to controlling kidney colonization by *L. interrogans*.

Reviewer #3: This manuscript presents an investigation into the role of the cGAS-STING signaling pathway in the host response to Leptospira interrogans infection. The authors demonstrate that Leptospira spp., although traditionally classified as extracellular bacteria, can be detected in the cytosol and within phagolysosomal compartments of macrophages. This intracellular presence allows for the sensing of leptospiral DNA by cGAS, followed by activation of the STING pathway and subsequent induction of type I interferon (IFN-I) responses. Using both in vitro infection of macrophages and in vivo infection models in C57BL/6 mice, the study shows that deficiency of cGAS or STING results in diminished IFN-I production, increased bacterial load, and enhanced renal colonization.

The experimental design is solid, and the data convincingly support the authors' conclusions. The manuscript is clearly written and well-organized. Overall, this is an important and well-executed study.

We thank the reviewer for their supportive comments.

Part II – Major Issues: Key Experiments Required for Acceptance

Reviewer #1: The conclusion of the authors is biased and without experimental foundation. Assuming that the pathways that were studied here are important for the host response, you would expect a change in outcomes. Other studies have shown, for example, that a dose of 2x10ˆ8 leptospires can cause death in mouse, specifically C57BL/6J. One would expect that with such important role and such a high dose of bacteria, knockout animals would become clinically ill or die. The difference in number of leptospires in the tissue is not enough to conclude that this pathways is “important” to control host infection.

Severity of disease in mice following infection with Leptospira sp. is highly dependent on the serovar and strain of Leptospira as well as host genetics. As mice can serve as a reservoir species, infection with *L. interrogans* serovar Copenhageni strain Fiocruz L1-130 leads to an asymptomatic infection leading to kidney colonization and prolonged urinary shedding of bacteria (PMID: 28270811; 24498450; 25032961). The reviewer is correct that certain knockout mice deficient in pathways such as TLR4 or MyD88 are susceptible to infection with Leptospira sp. and display increased mortality. However, the focus of our study was to determine innate immune factors that contributed to kidney colonization. As such defining that the cGAS-STING contributes to limiting renal colonization is highly important for understanding the innate immune response against Leptospira in reservoir species.

Figure 3H shows only a couple of animals that are outliers, with higher number of leptospires compared to wt animals. If there was an important role, you would expect to see the majority of the animals with higher number of leptospires in their kidneys.

We consistently saw statistically significant increased bacterial burdens in the kidneys of subcutaneously infected *Ifnar1*$^{-/-}$ and *Sting*$^{gt/gt}$ mice at 14 days post-infection. We have repeated this specific experiment to now include a total of 8 WT and 9 *Ifnar1*$^{-/-}$ mice pooled from three independent experiments and still observe significantly increased bacterial burdens in *Ifnar1*$^{-/-}$ mice compared to WT (Fig 3I). We have also independently examined the bacterial burden in the spleen and kidney of mice infected via the intraperitoneal route at day 14 post-infection. Consistent with the results of the low dose subcutaneous infection model we observed increased bacterial burdens in the kidneys of *Ifnar1*$^{-/-}$ (Fig 3G) and *Sting*$^{gt/gt}$ (Fig 5E) mice at day 14 after high-dose challenge. Taken together these data demonstrate a role for IFNAR and STING in limiting renal colonization with *L. interrogans*.

It is not clear why two different doses were used to infect animals for specific outcomes. It seems that both doses lead to colonization, so the choice to have two doses makes no sense, especially since a higher dose lead to no difference in leptospires in the kidneys, for example.

We apologize that we did not adequately explain the use of two models. The conventional model for mouse infections in the field has traditionally relied on infecting mice with a high dose of Leptospira intraperitoneally. However, this model is not necessarily very physiologic as mice are naturally infected via a cutaneous or mucocutaneous route after coming into contact with relatively smaller numbers of organisms shed in the urine from a colonized animal. The low-dose subcutaneous challenge model has been validated in previous studies (PMID 25411782; 19369528; 27031867). Hence, we utilized a low-dose subcutaneous infection model in which kidney colonization at late time-points could specifically be assessed. To help simplify comparisons between the two models we have now also examined bacterial burdens at day 14 post-infection in the spleens and kidneys of mice infected with the high-dose of *L. interrogans* intraperitoneally (Fig 3G and 5E). As described above, higher renal burdens were observed on day 14 in both the high-dose intraperitoneal and low-dose subcutaneous challenge models in *Ifnar1*$^{-/-}$ and *Sting*$^{gt/gt}$ mice.

In vitro experiments would be better interpreted if a non-pathogenic leptospires was used as control. It is unclear if all the effects seen here are due to pathogenic leptospires or simply leptospires in general, and that would be essential to take in consideration the conclusions and discussion established by the authors.

We agree that this is an important issue. As such we did evaluate the ability of the saprophytic *L. biflexa* Patoc I strain to drive the secretion of IFNβ from macrophages. We found that *L. biflexa* Patoc I was similarly capable of inducing IFNβ secretion compared to L. interrogans Fiocruz L1-130 (Supp Fig 1C).

Reviewer #2: Figures 2 are based on three biological replicates, which is insufficient to robustly support the claims regarding phagolysosomal trafficking kinetics in BMDMs during L. interrogans infection. This experiment needs to be repeated.

We have clarified these details in the methods section. Each biological replicate was done as a technical triplicate. The three biological replicates were pooled and expressed as the mean ± SEM. For Fig 2D the quantification was pooled from four independent experiments; in each experiment at least 3 fields with 15-40 cells per field were analyzed for colocalization. We have now also performed time-lapse confocal microscopy and found internalized bacteria colocalize with Rab5 confirming our earlier findings. These new data are included as Fig 2B.

In Fig 2A Cyto D does not show error bars.

In Figure 2A the curves are representative of three independent experiments, each performed as technical triplicates. The error bars were not visible as the symbols masked them. We have reduced the size of the symbols so error bars can be better visualized. The area under the curve data is pooled from three independent experiments and expressed as the mean ± SEM.

Moreover, cytochalasin D treatment data were not provided on IFN-α, and there is a lack of experimental validation for actin polymerization (e.g., phalloidin staining).

We attempted to assess IFNα levels using a commercial ELISA kit that detects IFN alpha2 and alpha4 subtypes (ThermoFisher #BMS6027) and were not able to measure detectable levels in the supernatants. Studies to assess cGAS-STING pathways frequently assess IFNβ alone (PMID: 27264171; 30804548; 35604097) due to these technical difficulties with measuring IFNα by ELISA. We have also validated that cytochalasin D did inhibit phagocytosis using pHrodo-labeled E. coli and phalloidin staining (Fig R1).

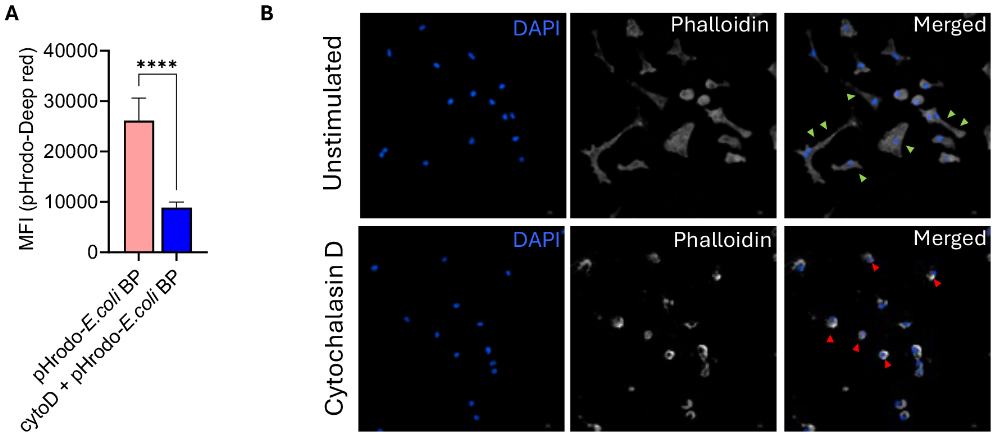

Fig R1. (A) Bone marrow derived macrophages (BMDM) were incubated with cytochalasin D (2μM) for 1h followed by 2h incubation with pHrodo-labelled *E. coli* bioparticles (Invitrogen, P35360). These bioparticles, labeled with a pH sensitive dye, fluoresce in the acidic pH environment of the phagosome but do not fluoresce at neutral pH. Following incubation, BMDMs were washed and stained with live/dead stain and analyzed by flow cytometry. Data are presented as the mean ± SEM pooled from three independent experiments. (B) BMDMs were incubated with cytochalasin D (2μM) and then visualized by immunofluorescence for phalloidin and counterstained with DAPI for cell nuclei. Unstimulated BMDMs display uniform phalloidin staining (green arrows). Upon addition of cytochalasin D, we observed a change in morphology with phalloidin restructuring as expected (red arrows). Images are representative of 3 frames collected from one experiment.

It would strengthen the study to include additional markers such as p65, Rab7, LAMP2, ORP, and LC3 to assess phagolysosomal and autophagic processes.

We agree that defining the phagolysosomal and autophagic processes during *L. interrogans* infection is of great importance. We have performed time-lapse confocal microscopy and found internalized bacteria recruited Rab-5 and were compacted into an intracellular vesicle. These new data are included as Fig 2B and 2C. We have also assessed LC3 and found no colocalization with *L. interrogans* in our preliminary studies (Fig R2). We are currently performing additional studies to fully define these processes. However, we feel that a detailed analysis of these pathways is beyond the scope of the current study.

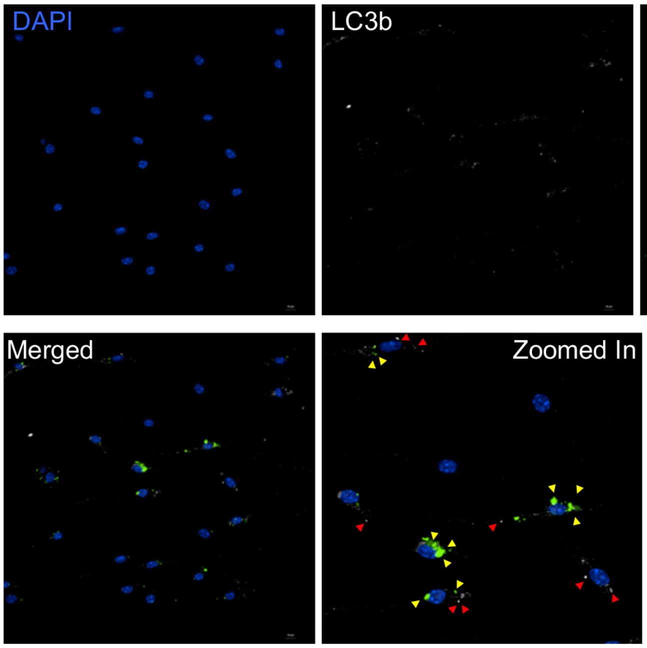

Fig R2. Bone marrow derived macrophages (BMDMs) were incubated with *L. interrogans* Fiocruz L1-130 (MOI 100) for 3h at 37°C. After incubation, cells were washed followed by fixing with 4% paraformaldehyde and permeabilized by triton X. The cells were stained for LC3b (white), bacteria (green), and nuclei with DAPI. It was observed that LC3b (red arrows) did not colocalizing with bacteria (yellow arrows). Images are representative of two experiments with three frames per experiment.

Results Fig 3. The description of these results needs to be clarified (add time points; Line 178: clarify ISGs, why is this expected), was IFN-alpha protein not tested? Were ISGs mRNA tested at 4h and 8h? If not significant state so in the results and show data in supplemental material. The figures need to represent clearly what was done (protein detection by ELISA, mRNA qPCR etc).

We apologize and have tried to clarify the description of this figure. Due to technical difficulties, we have been unable to detect measurable IFNα levels in culture supernatants by ELISA as described above. ISGs were assessed at 4h and 6h. ISGs were also lower in the *Ifnar1*$^{-/-}$ BMDM compared to WT at 4h post-infection; these data are provided here for the reviewer (Fig R3). We have edited the figure legends to ensure it is clear what assay is being performed.

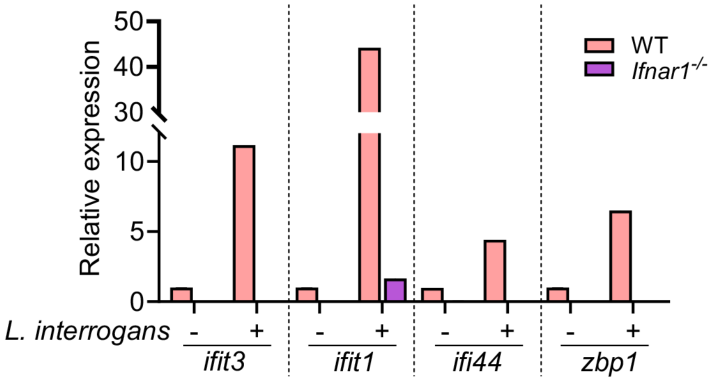

Fig R3. WT and *Ifnar1*$^{-/-}$ BMDMs were incubated with *L. interrogans* Fiocruz L1-130 (MOI 100) and the transcript levels of *ifit3, ifit1, ifi44,* and *zbp1* was assessed by qPCR. Data represents fold change calculated by ΔΔCt method and is shown as the mean from two independent experiments each performed in triplicate.

Fig 3F, it is unclear why the mRNA of ifna and ifnb is tested at 24h when the mice were euthanized at 3 dpi

As proinflammatory cytokines are induced early post-infection we chose to assess *Ifna* and *Ifnb* in the spleens of a separate cohort of mice at 24h post-infection.

The rationale for using different infection doses, animal numbers and routes between day 3 and day 14 experiments is unclear. Additionally, bacterial burden in the spleen, or IFN-α/β expression data at day 14, are not shown and would be important to support the conclusions. How this data supports the current claim of protecting kidney colonization needs to be clarified.

We apologize that this was not clear in the original manuscript. The conventional model for mouse infections in the field has traditionally relied on infecting mice with a high dose of Leptospira intraperitoneally. However, this model is not necessarily very physiologic as mice are naturally infected via a cutaneous or mucocutaneous route after coming into contact with relatively smaller numbers of organisms shed in the urine from a colonized animal. The low-dose subcutaneous challenge model has been validated in previous studies (PMID 25411782; 19369528; 27031867). Hence, we utilized a low-dose subcutaneous infection model in which kidney colonization at late time-points could specifically be assessed. To help simplify comparisons between the two models we have now also examined bacterial burdens at day 14 post-infection in the spleens and kidneys of mice infected with the high-dose of *L. interrogans* intraperitoneally (Fig 3G and 5E). As described above, higher renal burdens were observed on day 14 in both the high-dose intraperitoneal and low-dose subcutaneous challenge models in *Ifnar1*$^{-/-}$ and *Sting*$^{gt/gt}$ mice.

Results Fig 4. Was IFN-alpha protein not tested 4C and 4G? If no differences the data should be reported in this section and shown in supplemental material.

Due to technical difficulties, we have been unsuccessful at detecting measurable IFNα levels in culture supernatants by ELISA as discussed above.

Results Fig 5. The comments made above to Fig 3 apply to Fig 5. No significant differences were observed between wild-type and STING-deficient mice at day 3 post-infection. Although some differences emerge at day 14, the data are limited and insufficient to substantiate the

key claims. It is unclear why both spleen and kidney burdens are shown at day 3 but only kidney is assessed at day 14, and why different infection strategies were used across these timepoints. A clearer rationale is needed to justify expected differences at d3 and d14.

Please see explanation above.

Figure S1 does not show significant differences in IFN induction between pathogenic versus saprophytic Leptospira, or between live and heat-killed L. interrogans. These findings need to be better explained since the central claim that type I IFN signaling is specifically activated by pathogenic L. interrogans via the cGAS-STING pathway.

We have clarified this in the revised manuscript. These data suggest that both saprophytic and pathogenic strains of Leptospira are capable of inducing a type I IFN response from macrophages. Furthermore, heat-killed *L. interrogans* are also capable of driving a type I IFN response.

While the burden of Leptospira is higher in the kidneys of subcutaneous infected mice in the knockout (Sting1 and Ifnar1), the WT mice still have a substantial amount of Leptospira in their kidney. Is it fair to say, in the title, that IFN1 protects against renal colonization? Isn't it more accurate to say that it reduces colonization of the kidney?

The reviewer raises a good point. We have changed the title to "cGAS-STING dependent type I IFN reduces *Leptospira interrogans* renal colonization in mice."

Methods:
Line 333: what test was done to make sure dead Lepto remained dead? Re-growth?

We assessed growth of the heat-killed *L. interrogans* in culture 5 days later and confirmed that no viable bacteria were present.

Part III – Minor Issues: Editorial and Data Presentation Modifications

Reviewer #2: 1. Author Summary: the first paragraph is the same as in Abstract, it could be deleted and the message in this section can be more straight forward.

We appreciate the reviewer's comments and have edited this section to better fit as a non-technical summary.

2.The Introduction could be shortened to focus on how these studies advance the field. The first paragraph is redundant in the literature.

This section has been reduced in length and edited for clarity.

3. All figures: Captions need to be added to the figures for clarity. Regarding the clarity of the graphs, what does US mean in the axis X of graphs of Figure 1, 3, 4 and S1? Is it untreated samples? Is it possible to show the abbreviation in the legends?

We have edited the figures for clarity and stated the abbreviation for US (unstimulated) in the legend.

Reviewer #3: The statistical analysis of Figure 1 data was not indicated. Does the expression of TNF, IL10 and IL6 significantly increase after 4, 6 and 8 h?

Statistics for this figure have now been included.

I believe this study was previously approved by the institutional ethics committee. Please mention that and the number of this certificate.

This information is included in the Methods under “Ethics statement”

Please revise the format of references.

We have reformatted the references for PLOS Pathogens.
